# A Manual of Histology

**Published:** 1873-08-01

**Authors:** 


A Manual of Histology. By Prof. S. Stricker, American translation. Edited by Albert H. Buck, M.D. New York: Wm. Wood & Co. For sale by W. B. Keen & Cooke, Chicago.

It is difficult, in a book notice, to give a fair idea of this encyclopædia-like work of about eleven hundred pages.

The articles were written by different individuals, some of whom were only medical students, with, of course, their powers of observation imperfectly developed, and their acquaintance with the literature of their subjects meager. Others were written by men who have grown gray fighting the battles of science, and are as firmly fixed in their hobbies as German obstinacy and life-long defence of them can make one. The editor, Prof. Stricker, has given us the articles just as they were written, without comment, consequently the work has a very uneven and patch-work-like character, and numerous instances of conflicting views occur.

A marked characteristic of the work is the supercilious manner in which views differing from the author's are treated. Inquirers have a right to expect, in a work like the present, that all the modern views on histology, that are tenable, should be presented with fairness, and with some approach to fullness. But, throughout this work, views conflicting with the writer's are scarcely mentioned; and if they are mentioned, they are promptly disposed of by a peremptory denial of their correctness. Ewald Hering, for instance, gives no hint of the possibility of the existence of any opinion in regard to the anatomy of the liver differing from his own; and Gerlach, while using the methods of Lockhart Clarke for studying the spinal cord, never even mentions his name — unless, indeed, describing a column named after him be mentioning it.

A few of the writers, however, are more liberal. Von Recklinghausen, on the Lymphatics, is so liberal in his views, and weighs the arguments for and against his conclusions so candidly, as to win our confidence at once. The same might be said of Max Schultz, and some few others; and Stricker, in his account of them all, presents so many opposing views, and puts his own opinions forward so modestly, that we finish the article with an unsatisfied feeling. We like to have a writer lead our thoughts, even if we do not agree with him.

Many of the articles are deficient in illustrations. The drawings are so few and so poor in the series of articles on the intestinal Canal, that it is almost impossible to follow the descriptions. Pfluger's articles on the Salivary Glands, differing so greatly from all that we have heretofore believed, have not a single really convincing drawing to support them. The addition of more and better drawings would not only be an improvement in the clearness of the work (a direction, by the way, in which there is chance for improvement), but it would also allow the descriptions to be shortened; and would, besides, put us in some degree in the position of independent observers. But here, also, exceptions occur. The admirable articles of Max Schultz are profusely illustrated with carefully executed drawings, as is also the article by Recklinghausen, above referred to, and some others.

The value of most of the drawings is greatly diminished by omitting to state the magnifying power. Max Schultz's drawings have the magnify power put down in numerals, as have also some others; they are exceptions, however. The plan of attaching to each drawing a scale, copied from a micrometer magnified by the same glass as the drawing itself, costs so little trouble, and offers so many advantages, that it is strange that all microscopists do not use it. Such a plan is nowhere used in the book.

Taken as a whole, in spite of its faults, the work is an excellent one; it is carefully and conscientiously written, and is not only the best, but the only complete work on the subject in existence that is up to the times, and is indispensable to every one who would acquaint himself with the later advances of histology. L. C.

---

Trichinous Disease has been prevalent in Framingham, Mass.

